# Contraceptive use and associated factors among women seeking induced abortion in Debre Marko’s town, Northwest Ethiopia: a cross-sectional study

**DOI:** 10.1186/s12978-020-00945-4

**Published:** 2020-06-17

**Authors:** Lebeza Alemu, Yeshambel Agmus Ambelie, Muluken Azage

**Affiliations:** 1 Department of Midwifery, College of Health Sciences, Woldia University, Woldia, Ethiopia; 2 Department of Health Service Management and Health Economics, School of Public Health, Bahir Dar University, Bahir Dar, Ethiopia; 3 Department of Environmental Health, School of Public Health, Bahir Dar University, Bahir Dar, Ethiopia

**Keywords:** Contraceptive, Use, Factors, Induced abortion, Ethiopia

## Abstract

**Background:**

Contraceptive utilization is a practice that helps individuals or couples to avoid unwanted pregnancy. Even though there is the widespread availability of contraceptives, induced abortion remains an alarming public health problem in Ethiopia. Nationally, more than a third (35%) of women seeking an induced abortion service had a previous history of abortion. Therefore, this study aimed to assess the history of contraceptive use and identify associated factors among women seeking an induced abortion service in Debre Marko’s town, Ethiopia.

**Methods:**

An institutional-based cross-sectional study was conducted from March 15 to May 15, 2019. The sample size was 416 and each health institution was proportionally allocated based on the previous 2 months of patient flow. Systematic random sampling was used to select the study participants. A structured questionnaire was used to collect the data. Data were entered by EPI-data and analyzed using SPSS version 23. Bivariate and multivariable logistic regression analyses were carried out. Model fitness was assured.

**Results:**

The proportion of contraceptive use within the last 6 months before pregnancy was 41.3% among women seeking an induced abortion. Women who had good knowledge about contraceptives (AOR = 3.9; 95%CI: 2.36, 6.54), women who had a positive attitude about contraceptives (AOR=; 95%CI: 1.02, 2.56), women who had living children (AOR = 2.1; 95%CI; 1.04, 4.11), women who had frequent sexual practice (AOR = 2.5; 95% CI; 1.53, 4.21) and women discussed with their sexual partners about contraceptives (AOR = 1.9; 95%CI: 1.18, 3.18) were increase the odds of contraceptive use among women seeking an induced abortion.

**Conclusion:**

Contraceptive use among women seeking an induced abortion was low despite the expected national goal of 55% contraceptive use in 2020. Having good knowledge and having a positive attitude on contraceptives, and having a discussion on contraceptives with sexual partner were increase the odds of contraceptive use. The intervention should focus on abortion seeking women to achieve their contraceptive needs and encourage sexual partner discussion about contraceptives to improve joint partner collective decision-making.

## Plain English summary

Contraceptive utilization is practices that help individuals to avoid unwanted pregnancy to attain their desired children as their plan, and available methods of contraception should be customized to individual needs with a range of options. Despite widespread availability and awareness about family planning service induced abortion remains an alarming global public health problem due to poor contraceptive utilization. Therefore, this study aimed to assess the history of contraceptive use and identify associated factors among women seeking induced abortion since it is not well addressed in the study area.

All health institutions which provide legal abortion service in the study area were included. The sample size in each health institution was proportionally allocated based on the previous 2 months of patient flow, and systematically select the study participants.

The proportion of contraceptive use within the last 6 months before pregnancy was 41.3% among women seeking an induced abortion which is low despite the expected national goal of 55% contraceptive utilization in 2020. Having good knowledge and having a positive attitude about contraceptives; and having a discussion on contraceptives with a sexual partner were factors associated with contraceptive utilization. The intervention should focus on abortion seeking women to achieve their contraceptive needs.

## Background

Contraceptive use is a practice that helps individuals or couples to avoid unwanted pregnancy to attain their desired children as their plan, and available methods of contraception should be customized to individual needs with a range of options that are acceptable to all [1].

Nowadays, more attention is given on contraceptive use for multiple benefits as it prevents unintended pregnancies, which in turn reduces unsafe abortion and slows unsustainable population growth that affects the environment [2].

Worldwide in 2017, 63% of reproductive-aged women were using some form of contraception; and 58% of married or in-union reproductive-aged women were using a modern method of family planning, which comprises 92% of all contraceptive users [3].

The National Reproductive Health Strategy of Ethiopia emphasizes family planning to reduce unwanted pregnancies and enable individuals to achieve their desired family size by increasing access to and utilization of quality family planning services through building health infrastructure and the introduction of the health extension package [4].

Despite widespread availability and awareness about family planning service induced abortion remains an alarming global public health problem due to poor contraceptive utilization which is a personal and socioeconomic challenge for individuals, families, and society [5].

An estimated 620,300 induced abortions were done in Ethiopia in 2014. Between 2008 and 2014, the rate of abortions occurring in health facilities increases substantially from 27 to 53% [6]. The national figure of women receiving treatment due to complications of abortion was nearly doubled between 2008 and 2014, rising from 52,600 to 103,600 in Ethiopia [7].

Unintended pregnancy can be addressed by increasing availability and accessibility of modern contraceptives which in turn reduces the incidence of abortion with its related maternal death [8]. Despite many improvements, the non-use of contraceptives among women who wish to avoid pregnancy continues, which leads to unintended pregnancy and causing about 13% of abortions in Ethiopia [7].

Even though there is the widespread availability of contraceptives, induced abortion remains public health problem in Ethiopia. Nationally, more than a third of women seeking induced an abortion (35%) had had a previous history of abortion [9]. Therefore, this study aimed to assess the history of contraceptive use and identify associated factors among women seeking an induced abortion in Debre Marko’s town, Northwest Ethiopia.

## Materials and methods

### Study settings

An institution-based cross-sectional study was conducted from March 15 to May 15, 2019, among women seeking an induced abortion in Debre Marko’s town which is located at 300kms from Addis Ababa, capital city of Ethiopia. The town has one governmental referral hospital, 3 health centers, and two non-governmental organizations (NGO) clinics that give legal abortion services at the time of study period. The study population was women seeking an induced abortion services in the selected health institutions within the study period. Women having induced abortion due to obstetrical reasons were excluded from the study.

### Definitions

**History of contraceptive use:** use of any contraceptive method regularly with in the last 6 months before the current pregnancy time of conception.

**Induced abortion** refers to deliberate intervention to terminate the pregnancy.

**Good knowledgeable**: Refers study participants who answered ≥72% (mean score) of nine contraceptives knowledge questions.

**Poor knowledgeable**: Refers study participants who answered < 72% (mean score) of nine contraceptives knowledge questions.

**Positive attitude**: Refers study participants who answered ≥70% (mean score) of nine contraceptives attitude questions.

**Negative attitude**: Refers study participants who answered < 70% (mean score) of nine contraceptives attitude questions.

**Bad history of contraceptives:** A women who an experience of failure of contraceptive methods and/or got bad side effects with in the last 1 year before index pregnancy.

**Accessibility**: if the family planning service delivery center reached within 2 h (30 km) on foot was considered as access to family planning [10].

### Sample size determination and sampling procedures

EPI-info software was used to calculate the sample size using single proportion population formula by considering the following assumptions: p- contraceptive practice among women seeking abortion (*p* = 56.6%) [11]; margin of error (5%) and 95% CI (z_1/2_ = 1.96). The final sample size was 416 by considering 10% non- response rate.

**Fig. 1:**
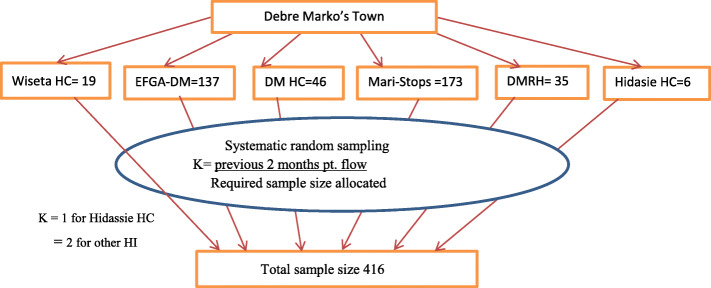
Sampling procedures, Debre Marko’s, Ethiopia, 2019

All health institutions which provide legal abortion service in the study area were included. The sample size for each health institution was determined proportionally based on the previous 2 months of clients flow. Systematic random sampling was used to select the study participants (Fig. 1). The k^th^ interval of systematic sampling was determined by dividing the previous 2 months client flow with the required sample size from each health institutions.

### Data collection instruments and procedures

Interviewer administered questionnaire was used to collect the data. The questionnaires initially prepared in English and translated to Amharic and again back to English to check the consistency. The questionnaire constitutes socio-demographic variables, reproductive and sexual characteristics, health institution accessibility, knowledge and attitude questions on contraceptive and contraceptive use question. The questionnaire was filled by the health professionals who were working in the same health institution out of maternal and child health room. One BSc Midwife was assigned as a supervisor who supervises the data collection throughout the process in each health institution.

Data quality was assured using pre-testing and training of data collectors and supervisors. The topics of the training were data confidentiality, responders’ right, informed consent, the objective of the study, on the techniques of the interview and filling the questionnaire.

### Data analysis

Data were entered using EPI data and analyzed using SPSS version 23. Descriptive statistics were used to describe the data. Bivariate analysis was employed to examine the association between dependent and independent variables. Finally, multivariable logistic regression was done to determine the independent effect of each factor and control confounding effect. A *p*-value < 0.05 was used as cut of point to declare statistically significant. Multicollinearity was checked to see the correlation among independent variables. Model fitness was checked with Hosmer Lemshow test. Knowledge questions were categorized and coded as (good = 1 and poor = 0) whereas attitude questions were categorized and coded as (1 = Positive attitude and 0 = Negative attitude). Cronbach alpha was checked to assure the internal consistency of attitude measuring questions.

## Results

### Socio-demographic characteristics

A total of 416 women participated in this study with a response rate of 95.4%. The mean age of the participants was 23.23(SD ± 4.6) years with a predominant age group of 20–24 years (48.1%). The majority of the participants were Orthodox religious followers (87.9%) and urban residents (79.1%). Most of the participants were not married (64.7%) and completed secondary education (34.0%) (Table 1).

**Table 1:** Socio-demographic characteristics of women seeking an induced abortion, Debre Marko’s town, Ethiopia, 2019(*n* = 397)

Variables	Frequency	Percentage
Age of participants		
15–19	80	20.2
20–24	191	48.1
25–29	67	16.9
> =30	59	14.9
Residence		
Urban	314	79.1
Rural	83	20.9
Religion		
Orthodox	349	87.9
Muslim	25	6.3
Protestant	18	4.5
Others	5	1.3
Marital status		
Married	105	26.4
Unmarried	257	64.7
Divorced/Widowed	35	8.8
Educational status		
No formal education	56	14.1
Primary	100	25.2
Secondary	135	34.0
Tertiary & above	106	26.7

### Obstetrics, sexual and reproductive characteristics

The majorities of the participants have no living children (76.1%) and were at first trimester with the current pregnancy (94.7%). The majority of the participants had no sexual partner discussion about contraceptives (67.5%) and they had no previous bad history of contraceptive utilization (79.1%). Most of the participants were accessible for contraceptive service (95.2%). The mean age of first sex was 18.3 years with (SD ± 2.6) years (Table 2).

**Table 2:** Sexual and Reproductive characteristics of women seeking an induced abortion, Debre Marko’s town, Ethiopia, 2019(*n* = 397)

Variables	Frequency	Percentage
Number of living children		
0	302	76.1
> =1	95	23.9
Age of first sex		
< =14	29	7.3
15–19	219	55.2
> =20	149	37.5
Gestational age		
1st trimester	376	94.7
2nd trimester	19	4.8
3rd trimester	2	0.5
Sexual partner discussion		
No	268	67.5
Yes	129	32.5
Bad history of contraceptive utilization		
No	314	79.1
Yes	83	20.9
Accessibility of FP service		
No	19	4.8
Yes	378	95.2

### Knowledge and attitude about contraceptives

More than half of the participants had good knowledge about contraceptives (55.4%) and around half of the participants had a positive attitude for contraceptives (50.1%) (Table 3).

**Table 3:** Knowledge and attitude about contraceptives of women seeking an induced abortion, Debre Marko’s town, Ethiopia, 2019(n = 397)

Variables	Frequency	Percentage
Knowledge about contraceptives		
Poor knowledge	220	55.4
Good knowledge	177	44.6
Attitude for contraceptives		
Negative attitude	198	49.9
Positive attitude	199	50.1

### History of contraceptive use

**Fig. 2:**
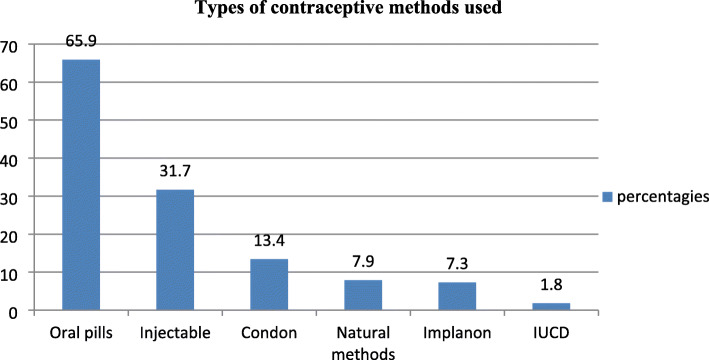
Types of contraceptive used among abortion seeking women in Debre Marko’s town, Ethiopia, 2019(*n* = 164)

Among participants, 164(41.3%) reported that they had used contraceptives within the last 6 months before their current pregnancy (Table 4). Among the participants, 9.1% had pregnancy while using contraceptives (failure rate). Among contraceptive users, 65.9% of them used oral contraceptives, and 31.7% used injectable contraceptives (Fig. 2).

**Table 4:** Prevalence history of contraceptive use among women seeking an induced abortion, Debre Marko’s town, Ethiopia, 2019

	Frequency	Percentage
History of contraceptive Use		
No	233	58.7(53..9–63.5)
Yes	164	41.3(36.5–46.1)
Contraceptive use at the time of Conception (failure rate)		
No	361	90.9
Yes	36	9.1
Intention to Post abortion contraceptive utilization		
No	28	7.1
Yes	369	92.9

### Factors associated with the history of contraceptive use

In multivariable logistic regressions, participants’ knowledge and attitude about contraceptives, number of living children, frequency of sexual practice, and having discussions with sexual partners about contraceptives were found significantly associated with contraceptive use. The odds of contraceptive use among women who had good knowledge about contraceptive were 3.9 times (95%CI: 2.360, 6.548) higher compared to their counterpart. The odds of contraceptive use among women who had a positive attitude about contraceptives were 1.6 times (95%CI: 1.023, 2.561) higher compared to women who had a negative attitude.

Women who had living children were 2.1 times (95%CI; 1.040, 4.112) higher the odds of contraceptive use compared to those who had no children. The odds of contraceptive use among women who had frequent sexual practice were 2.5 times (95% CI; 1.538, 4.213) higher compared to women who had an infrequent sexual practice. Lastly, women who had a discussion on contraceptives with their sexual partners were found 1.9 times (95%CI: 1.185, 3.181) higher odds of contraceptive use than their counterparts (Table 5).

**Table 5:** Bivariate & multivariable regression of women seeking an induced abortion, Debre Marko’s town, Ethiopia, 2019(n = 397)

	Contraceptive utilization		COR(95%CI)	AOR(95%CI)	P-value
	No	Yes			
Age					.113
15–19	52	28	1	1	
20–24	109	82	1.397(.813, 2.401)	.682(.363, 1.282)	.237
25–29	35	32	1.698(.874, 3.298)	.592(.260, 1.347)	.224
≥ 30	37	22	1.104(.549, 2.223)	.294(.110, .782)	.015
Marital status					
Married	40	65	3.545(1.569, 8.011)	1.860(.704, 4.917)	.211
Unmarriec	169	88	1.136(.532, 2.426)	1.127(.428, 2.963)	.809
Divorced	24	11	1	1	
Number of living children					
0	117	185	1	1	
≥ 1	47	48	1.548(.973, 2.463)	2.068(1.040, 4.112)	.04
Knowledge					
Poor	36	134	1	1	
Good	128	99	4.813(3.063, 7.562	3.931(2.360, 6.548)	.000
Attitude					
Negative	67	131	1	1	
Positive	97	102	1.859(1.240, 2.787)	1.619(1.023, 2.561)	.041
Sexual partner discussion					
No	86	182	1	1	
Yes	78	51	3.237(2.092, 5.007)	1.941(1.185, 3.181)	.009
Frequency of sexual practice					
Infrequent	192	92	1	1	
Frequent	41	72	3.665(2.321, 5.788)	2.545(1.538, 4.213)	.000
Residence					
urban	170	144	2.668(1.540, 4.624)	1.51(0.807, 2.836)	.019
Rural	63	20	1	1	

Variable(s) entered on step 1: SPAge, Residenc, MaritalStat, Educational status, Number of living children, Frequency of sexual practice, Knowledge, Attitude, History of abortion, Disscussion with sexual partner, Accessibilty, Bad history of Contraceptive utilization

**Fig. 3:**
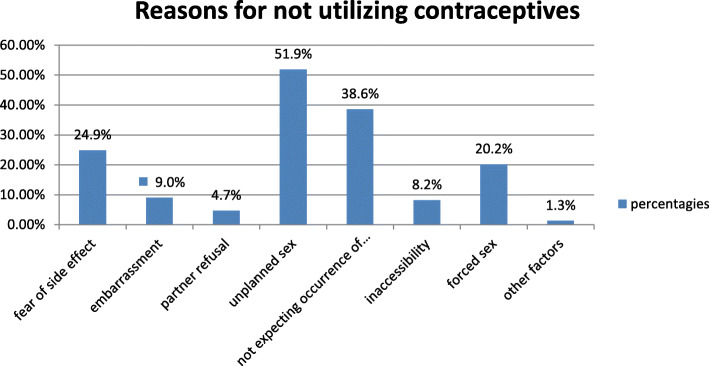
Reasons for not using contraceptives among women seeking induced abortion, Debre Marko’s town, Ethiopia, 2019(*n* = 233)

Study participants reported that unplanned sex was the main reason for not using contraceptives before their current pregnancy. About 51.9% of the participants reported that their pregnancy was due to unplanned sex. The study also showed that the other reason for none contraceptive use before the current pregnancy was not expecting the occurrence of pregnancy (38.6%). The majority of the participants also raised hormonal side effects of the contraceptives as their reason for none contraceptive use (Fig. 3).

## Discussion

The result of this study revealed that only 41.3% (95%CI: 36.5–46.1) of abortion seeker women were utilized contraceptives within the last 6 months before their index pregnancy. Even though the women had access to contraceptives, they didn’t use a contraceptive to prevent their unplanned pregnancy which leads them to abortion-related problems and needs a high national cost for abortion service.

This finding is lower than the studies done in Addis Ababa (56.6%) and Hawassa (66%) among women seeking an induced abortion. The possible reasons may be due to the time variation of contraceptive use before the index pregnancy and the frequency of abortion. For example, in the current study shows contraceptive use within the last 6 months before the index pregnancy, regardless of the frequency of abortion, whereas the studies done in Addis Ababa and Hawassa reported ever use of contraceptives before the index pregnancy. Moreover repeated abortion seeking women who had more likely to use contraceptives who were included in Addis Ababa study’s [11, 12].

Similarly, the result of this study is lower than the studies done in India (59.1%), Iran (63.5%) and Yorkshire **(**US) (69%). The possible reasons may be due to those studies show ever uses of contraceptives before the current pregnancy. This implies that a woman may have higher contraceptive utilization experience before the index pregnancy regardless of the time-bound [13–15].

This finding is in line with the study done in China, which shows 38% of the participants who received abortion service were utilizing any contraceptive during the 6 months preceding the survey [16]. The current result also supported by the study done in Missouri(US) and Cape Town (South Africa) as they revealed that 37 and 44.1% of the study participants reported utilized contraceptives before the current pregnancy respectively [17, 18].

On the contrary, the current finding is higher than the studies done in Bangalore and Pakistan, which reported that 35.7 and 27% of the participants had contraceptive use experience before the index pregnancy respectively. This variation may be due to participant’s contraceptives knowledge difference since 23.8% in Bangalore and 17% in Pakistan lacking awareness on any contraceptive methods as lack of knowledge is the biggest hurdle the practice of contraceptives. There was also in-laws opposition to use contraceptives in Pakistan since 22% of women raised it for the reason of none contraceptive use [19, 20].

The finding of the current study revealed that women who had living children were increase the odds of contraceptive use compared to those women had no living children which is consistent with the study done in North Showa Zone, Ethiopia. The possible explanation could be due to the fact that women who had living children might have a lower future fertility desire and their intention to use contraceptive methods might be high [21]. The finding of this result is also in line with the study done in Western Cape (South Africa), which revealed that women who have living children were more likely to use contraceptive due to less chance of sexual practice since they may be unmarried (58). This finding is similar to previous reports from Bangladesh and Tanzania which reported that as the number of living children increases, the use of modern contraceptives increases [22, 23].

Contraceptive use among women seeking abortion who have frequent sexual practice had more likely than women who had an infrequent sexual practice. The possible reason may be related to the perception of women who had reduced sexual exposure and reduced fecundity could have low risk of pregnancy and lead them use of contraceptive is unnecessary [24].

The finding of this study showed that women who had good knowledge about contraceptive was one of the predictors of contraceptives use which is consistent with the studies done in Ethiopia; Dabat [25], Fogera [26] and abroad Sweden [27]. It is evident that lack of basic knowledge on contraceptive is the biggest hurdle in the practice of contraceptives [28].

Women who have a positive attitude about contraceptives had a better contraceptive history than women who have a negative contraceptive attitude which is supported by the studies done in different parts of Ethiopia as it showed women who had a favorable attitude towards FP were more likely to practice contraceptives [29].

In this study, women who had a discussion with their sexual partner about contraceptives had a better use of contraceptive history, which is consistent with other studies done in Bahir Dar and North Showa, Ethiopia. These studies revealed that women who had a discussion on contraceptive methods with sexual partner were more likely utilize contraceptives since male involvement encouraged their sexual partners to use contraceptives regularly [21, 30].

This study has its own limitations. Social desirability bias is one of the limitations of this study since women may report more acceptable response. Recall bias might be also occurred since women may forget the exact their current pregnancy time of conception. This study did not establish cause and effect relationship between independent and outcome variable due to the limitation of cross-sectional study design.

## Conclusions

Contraceptive use among women seeking an induced abortion was low despite the expected national goal of 55% contraceptive use in 2020. Women’s marital status, knowledge about contraceptives, and sexual partner discussion of contraceptives were significantly increasing the use of contraceptives.

The identified factors help to strengthen the existing country’s strategies to focus on abortion seeking women to decrease the rapid increment of abortion practice and its related health and economic burden. Adequate information and instructions on contraceptive methods should be given for the users to improve women’s contraceptive knowledge. Women and their sexual partner should be encouraged to discuss on importance of contraceptive use to improve joint partner collective decision-making. In this study, contraceptive failure was also occurred among majority of women who used emergency contraceptive which could be an area of further research.

## Data Availability

The datasets used and/or analyzed during the current study are available from the corresponding author on reasonable request.

## Abbreviations

AOR: Adjusted odds ratio
CI: Confidence interval
COR: Crude odds ratio
FP: Family planning
HSTP: Health sector transformation plan
NGO: None Governmental Organization
SD: Standard deviation
SNNP: South Nation Nationality and People
SPSS: Statistical package for the social sciences
USA: the United States of America
WHO: World Health Organization
